# Sodium Humate Alleviates Enterotoxigenic *Escherichia coli*-Induced Intestinal Dysfunction via Alteration of Intestinal Microbiota and Metabolites in Mice

**DOI:** 10.3389/fmicb.2022.809086

**Published:** 2022-03-25

**Authors:** Dong Wang, Yanjun He, Kexin Liu, Shouxiang Deng, Yuying Fan, Yun Liu

**Affiliations:** Heilongjiang Key Laboratory of Experimental Animals and Comparative Medicine, College of Veterinary Medicine, Northeast Agricultural University, Harbin, China

**Keywords:** sodium humate, mice, enterotoxigenic *Escherichia coli* K88, intestinal barrier integrity, intestinal microbiota, metabolome

## Abstract

Enterotoxigenic *Escherichia coli* (ETEC) can damage intestinal epithelial barrier function and lead to serious intestinal diarrhea in newborns and young animals. Sodium humate (HNa) is natural organic bioactive compound possessing antibacterial, anti-inflammatory, and anti-diarrheal properties. This study investigated the alleviative potential of HNa on the impaired intestinal barrier and intestinal inflammation, and regulatory effects on gut microbiota and metabolites in ETEC K88 infected mice. A total of 30 female mice were randomly assigned into three groups. The mice in the control and ETEC groups were gavaged with 0.2 mL of sterile saline, while the mice in the ETEC + HNa group were gavaged with 0.2 mL of 5% HNa, daily. On day 8, the mice in ETEC and ETEC + HNa group were challenged with ETEC K88. The trial lasted for 12 days. HNa administration elevated ETEC K88-induced body weight loss and ameliorated jejunum and colon pathological injury. HNa also reduced the levels of pro-inflammatory cytokines in the serum, jejunum, and colon. Additionally, HNa reduced intestinal barrier damage by up-regulating the expression of tight junction proteins (TJPs) and mucosal repair factors. 16s rDNA gene sequencing results showed that HNa increased the abundance of beneficial bacteria *Lactobacillus, Prevotella_9*, and *Odoribacter* but decreased the abundance of pathogenic bacteria *Escherichia* and *Gastranaerophilales* in the feces of mice. Moreover, metabolomic analysis revealed that the concentrations of 15 metabolites, the pathways of protein digestion and absorption, and propanoic acid metabolism were changed by HNa administration. In conclusion, HNa could alleviate ETEC K88-induced intestinal dysfunction through restoring intestinal barrier integrity, modulating gut microbiota, and metabolites.

## Introduction

It is well known that *Escherichia coli* (*E. coli*) can cause severe intestinal diarrhea in humans and animals (Jesser and Levy, 2020). Enterotoxigenic *Escherichia coli* (ETEC) K88 is the most common serotype of ETEC which can induce intestinal disease and diarrhea in newborns and young animals (Yu et al., 2020). Intestinal barrier integrity plays an important role in protecting the gut against pathogenic microorganisms (Viennois et al., 2020). ETEC could damage intestinal barrier function, and induce inflammatory response by colonizing in intestinal epithelium and then secreting enterotoxins, ultimately inducing inflammatory bowel disease (Ren et al., 2017). In addition, ETEC infection following body weight loss and high mortality resulted in considerable economic losses in livestock industry (Becker et al., 2020). Antibiotics have long been used to prevent and treat intestinal diseases in animals. However, the growing of antibiotic resistance and negative public health outcomes has gained worldwide concern (Haiwen et al., 2019). Moreover, a recent study demonstrated that long-term use of antibiotics had detrimental effects on intestinal barrier function (Wang Y. et al., 2020).

Humic acids (HAs) derived from decaying organic matter in the peat and sapropel possess various pharmacological properties, thus have been widely used in traditional Chinese medicine (Pavlovska et al., 2020). *In vitro* and *in vivo* toxicological trials have shown that appropriate dose of HAs have no genotoxicity (Murbach et al., 2020). As a salt of HAs, sodium humate (HNa) has been used as a therapeutic agent for dyspepsia, diarrhea, and acute intoxication in animals with potential anti-microbial, antivirus, anti-inflammatory, antidiarrheal as well as anti-rheumatic properties (Brandts et al., 2021). An earlier study showed that dietary HNa supplementation decreased the *E. coli* adhesion and colonization in the gut of ETEC infected piglets, indicating its underlying antimicrobial activity (Kaevska et al., 2016). HNa inclusion also improved the growth performance, immune function, antioxidant capacity, and decreased diarrhea incidence in weaned piglets (Wang Q. et al., 2020). These results provide evidences that HNa may protect against *E. coli* infection in animals. However, the mechanism of action remains unclear. Therefore, the objective of this study is to investigate the therapeutic effects and the mechanism of action for the oral administration of HNa on ETEC K88-infected mice by determining intestinal morphology, intestinal barrier integrity, inflammatory responses, intestinal microbiota as well as metabolites.

## Materials and Methods

Sodium humate (purity, 75%) was provided by the Institute of Coal Chemistry, Chinese Academy of Sciences (Shanxi, China). It consists of 75% humic acid (dry basis), 20.52% burning residue (dry basis), 14.22% water (air dry basis), and 4.48% water soluble substances (dry basis).

The *E. coli* strain ETEC K88 was maintained in our laboratory. ETEC K88 was cultured in Luria–Bertani broth at 37°C for 8 h and centrifugated at 5,000 g for 10 min until modeling. Cells were washed with phosphate buffer saline (PBS) three times. Finally, ETEC K88 concentration was adjusted to 5 × 10^10^ colony-forming unit (CFU) mL^–1^ in PBS.

### Animals, Experimental Design, and Sample Collection

The experimental protocol was approved by the Ethics Committee of Northeast Agricultural University (Harbin, China). A total of 30 female mice (5 weeks old) were purchased from Chang Sheng Biotechnology Co., Ltd. (Liaoning, China) and housed under constant temperature and humidity, with a 12 h light/dark cycle. All mice had free access to feed and water throughout the experimental period. After 7 days of adaption period, all of the mice were randomly assigned to three groups (*n* = 10), including control group, ETEC group, and ETEC + HNa group. The trial lasted for 12 days. Mice were fasted for 12 h and water for 4 h before ETEC K88 infection. One hour prior to ETEC K88 infection, mice received cimetidine (50 mg/kg; Abbott Laboratories, Chicago, IL, United States) intraperitoneally to reduce the effect of stomach acid on the bacterial organisms. The mice in the control and ETEC groups were gavaged with 0.2 mL of sterile saline, while the mice in ETEC + HNa group were gavaged with 0.2 mL of 5% HNa, daily. On day 8, the ETEC and ETEC + HNa groups were challenged with 0.2 mL of 5 × 10^10^ CFU mL^–1^ ETEC K88 by intragastric administration (Yu et al., 2020). In the previous study, we investigated the bacteriostatic effects of 0, 1, 3, and 5% of HNa *in vitro* and vivo, and found that 5% of HNa showed the best bacteriostatic activity. Therefore, we used 5% of HNa in the present study (Wang D. et al., 2020).

The mice were weighed every day, individually. Feces scoring procedure was performed as follows: 0 = normal stool; 1 = color change/consistency; 2 = presence of wet tail or mucosa; 3 = liquid stools. A score of 1 was considered diarrhea. On day 13, blood samples were collected from the orbital venous plexus and centrifuged for 15 min at 3,000*g*, 4°C. The serum supernatants were collected and stored at −20°C for further analysis. Subsequently, the mice were sacrificed by cervical dislocation, then the spleens were weighed immediately to calculate the spleen index. Spleen index (%) = spleen weight (g)/body weight (g). The fresh feces, jejunum, ileum, colon, liver, and spleen tissue were collected for bacterial colonization analysis. The jejunum and colon samples were flushed with PBS and then approximately 1 cm of middle segments was cut off and fixed in 4% paraformaldehyde solution for histomorphology analysis. Additionally, no more than 1 mm^3^ fresh jejunum and colon tissue block was separated and fixed with 2.5% glutaraldehyde at 4°C for transmission electron microscopy analysis. Another jejunum and colon samples were quickly frozen in liquid nitrogen and stored at −80°C for RNA and protein extraction. Fresh feces were collected and stored at −80°C for further analysis of microbiota and metabolites.

### Bacterial Colonization

Each fresh fecal sample, jejunum, ileum, colon, liver, and spleen tissue was homogenized in sterile PBS. Ten-fold serial dilutions of homogenates were then plated on MacConkey (OXOID, Hampshire, United Kingdom) plates. Then, the MacConkey agar plates were incubated for 12 h at 37°C under aerobic conditions. The results are expressed as log_10_ CFU/mg of feces or tissues. All counts were performed in triplicate. Colonies growing on the MacConkey agar plates were considered to be enterobacteria-like bacteria belonging to the family *Enterobacteriaceae*.

### Histomorphological Analysis

The jejunum and colon fixed in 4% paraformaldehyde were embedded in paraffin, and then sliced into 5 μm-thickness slices, which were deparaffinized in xylene and stained with hematoxylin/eosin (H&E). The tissue was examined using light microscopy (Leica Microsystems, Wetzlar, Germany). The colon pathology was evaluated by a single-blinded scorer using a validated scoring system. The villus height and crypt depth of jejunum and the length, histologic damage score, and crypt depth of colon were measured with Image-Pro Plus 6.0 software (Media Cybernetics, Rockville, MD, United States).

Alcian blue periodic acid Schiff (AB-PAS) staining was used to detect goblet cells density and mucus layer thickness in the jejunum and colon. Fixed intestinal tissues were embedded in paraffin. After deparaffinization, the tissue sections were stained with PAS. The mucus layer thickness and quantities of goblet cells were counted by Image-pro Plus 6.0 software.

The jejunum and colon tissues were fixed with 2.5% glutaraldehyde for 1 h and then fixed with 1% osmium tetroxide for 2 h at room temperature in the dark, followed by dehydration with grade ethanol for 10 min each, two times. The samples were then embedded in resin and cut to 50–60 nm on ultramicrotome (Leica UC7; Leica Microsystems GmbH, Wetzlar, Germany). Then the ultra-thin sections were stained with 0.25% lead citrate and 2% uranyl acetate. The ultrastructure of intestinal samples was observed under a transmission electron microscope (H-7650, Hitachi, Japan) at an accelerating voltage of 80 kV.

### Enzyme-Linked Immunosorbent Assay

The concentration of diamine oxidase (DAO), D-lactate (D-lac), and lipopolysaccharide (LPS) in the serum, interleukin-1β (IL-1β), interleukin-6 (IL-6), interleukin-10 (IL-10), and tumor necrosis factor-α (TNF-α) in the serum, jejunum, and colon were determined using the commercial ELISA kits (Jingmei Biotechnology Co., Ltd, Jiangsu, China) according to the manufacturer’s instructions.

### Immunohistochemistry

Paraffin-embedded slides from jejunum and colon tissues were deparaffinized in dimethyl benzene and graded ethanol. After deparaffinization, tissue sections were blocked with 5% BSA and stained with primary antibodies: occludin, claudin-1, and zona occludens 1 (ZO-1) (1:200, Wanlei Biotechnology, Liaoning, China) overnight at 4°C. Sections were washed three times with PBS and treated with HRP labeled goat anti-rabbit IgG secondary antibody, then incubated at room temperature for 20 min and washed three times with PBS. Subsequently, diaminobezidin (DAB) substrate color liquid was added to sections. All images were captured with microscope (Nikon DS-U3, Nikon, Tokyo, Japan) and analyzed using Image-Pro Plus 6.0 software.

### Real-Time Polymerase Chain Reaction for Gene Expression Analysis

Total RNA from jejunal and colon tissue was isolated by Trizol reagent (TaKaRa Biotechnology, Dalian, China). The concentration and purity of extracted RNA were measured by microspectrophotometer (NanoDrop-1000, Thermo Fisher Scientific, Waltham, MA, United States). Then PrimeScriptTMRT reagent Kit (TaKaRa Biotechnology, Dalian, China) was used to produce the complementary DNA. qRT-PCR procedure was carried out to determine the mRNA abundance of Mucin-1, Mucin-2, Mucin-3, transforming growth factor-β1(TGF-β1), proliferative cell nuclear antigen (PCNA), epidermal growth factor receptor (EGFR), occludin, claudin-1, ZO-1, and beta-actin (β-actin) using ChamQ SYBR qPCR Master Mix Kit (Vazyme Biotechnology, Nanjing, China) based on Applied Biosystems 7500 Real-time PCR System (Life Technologies, Carlsbad, CA, United States). The relative mRNA abundance of the target genes was calculated using the 2^–ΔΔ*Ct*^ method and normalized with expression level of endogenous reference gene (β-actin). The qRT-PCR primers for target genes were commercially synthesized by Sangon Biotechnology (Shanghai, China) and listed in Supplementary Table 1.

### Western Blot Analysis

Frozen jejunum and colon tissue samples were homogenized in RIPA lysis buffer containing protease inhibitor. The total protein concentration was determined using a BCA protein assay Kit (Beyotime Biotechnology, Shanghai, China). Approximately 30 mg of protein were loaded and separated by SDS-PAGE and then transferred onto polyvinylidene difluoride (PVDF) membranes. The membranes were blocked with 5% skimmed milk for 2 h at room temperature and then incubated with primary antibodies: PCNA, EGFR, and TGF-β1 (1:750) (Wanlei Biotechnology, Liaoning, China) overnight at 4°C, and then incubated with secondary antibody for 1 h at room temperature. The expression of target proteins was detected with ECL chemiluminescence reagents (E412-01; Vazyme Biotechnology, Nanjing, China) and Imager-Bio-Rad (Bio-Rad Laboratories, Inc., Hercules, CA, United States), then quantified using Image J software.

### DNA Extraction, 16S rDNA Amplification, and High-Throughput Sequencing

Total genome DNA was extracted from fecal samples using cetyltriethylammnonium bromide (CTAB) method, and then the integrity of extracted DNAs was detected by 1% agarose gel. The V3–V4 regions of the bacterial 16S rDNA gene were amplified by specific primers: 341F (5′- CCTACGGGNGGCWGCAG -3′) and 806R (5′- GGACTACHVGGGTATCTAAT -3′) with the following procedures: initial denaturation at 98°C for 1 min, followed by 30 cycles of denaturation at 98°C for 10 s, annealing at 50°C for 30 s, and elongation at 72°C for 60 s, finally 72°C for 5 min. The amplicons were then purified and pooled in paired-end sequence on an Illumina MiSeq platform (Illumina, San Diego, CA, United States).

Microbiome raw data was analyzed using the QIIME2 platform (version 2019.4). Briefly, raw reads were quality filtered, assembled, and chimeric sequences were removed using DADA2 plugin, which generated unique amplicon sequence variants (ASVs). Subsequently, we used the SILVA reference database classifier (version 138) for the classification of ASVs. Determinations of alpha and beta diversities were also conducted in QIIME 2. Principal co-ordinates analysis (PCoA) plots were generated using the “ggplot2” packages of the R software (version 3.3.1). Also, we performed permutational multivariate analysis of variance based on Bray–Curtis distances using the Anosim function in the package “vegan” in R software (version 3.3.1). Differentially abundant genera between groups were identified using linear discriminant analysis effect size (LEfSe) and linear discriminant analysis (LDA).

### Fecal Metabolites Assessment

Fecal samples were prepared according to previously described procedures (Hu et al., 2020). LC-MS/MS analyses were performed using an UHPLC (1290 Infinity LC, Agilent Technologies, Palo Alto, CA, United States) coupled to a quadrupole time-of-flight (TripleTOF 6600, AB Sciex, United States) in Shanghai Applied Protein Technology Co., Ltd. For HILIC separation, samples were analyzed using a 2.1 mm × 100 mm ACQUIY UPLC BEH Amide 1.7 μm column (Waters, Milford, MA, United States). In both ESI positive and negative modes, the mobile phase contained A = 25 mM ammonium acetate + 25 mM ammonia water and B = acetonitrile.

The collected data were used to identify the structure of metabolites using self-built MetDDA and LipDDA methods (Shanghai Applied Protein Technology Co. Ltd, Shanghai, China). The original data were converted into mzXML format by ProteoWizard MSConvert. After normalized, the processed data were analyzed by R package (ropls). The 7-fold cross-validation and response permutation testing was used to evaluate the robustness of the model. The variable importance in the projection (VIP) value of each variable in the orthotopic partial least-squares discriminant analysis (OPLS-DA) model was calculated to indicate its contribution to the classification. Metabolites with the VIP value >1 were further applied to Student’s *t*-test at univariate level to measure the significance of each metabolite, and the significance was declared at *P* < 0.05. Later, the differential metabolites were further conducted to cluster analysis and metabolic pathway analysis by KEGG^1^. Fisher’s Exact Test was used to investigate the significance level of the enrichment pathway.

### Statistical Analysis

All of the data were presented as means with their standard errors (SEM). Significance level of differences between two groups was analyzed using Student’s unpaired *t*-test, while multiple comparisons were analyzed by one-way ANOVA (SPSS 20.0, Inc., IBM, Chicago, IL, United States) followed by Tukey’s test. Differences were considered significant at *P* < 0.05. The correlation analysis among intestinal microbiota and metabolites was estimated by Spearman’s correlation coefficient. Correlations were considered significantly different at *r* > 0.50 or *r* < −0.50, *P* < 0.05.

## Results

### Clinical Symptoms and Jejunum and Colon Morphology

**FIGURE 1:**
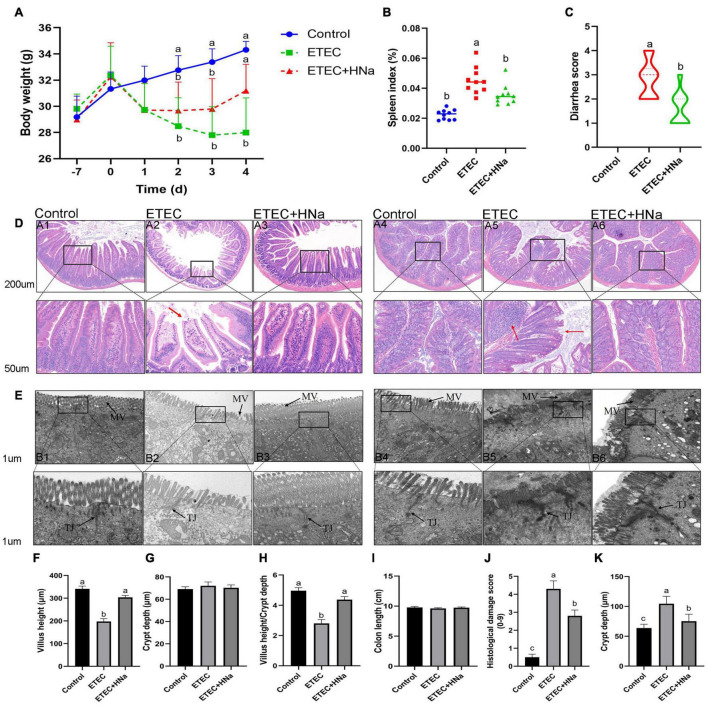
Effects of sodium humate on clinical symptoms, jejunal, and colon pathological morphology of ETEC K88-infected mice. **(A)** Body weight change, days –7 to 0 were pre-infection, and days 0–4 were post-infection. **(B)** Spleen index of mice. **(C)** Diarrhea score. **(D)** Representative images of the jejunum (A1–A3) and colon (A4–A6) stained with H&E (200 μm). The red arrow points to the damaged jejunum and colon villus. **(E)** Jejunum (B1–B3) and colon (B4–B6) morphology shown by transmission electron microscopy (1 μm). MV, microvilli; TJ, Tight junction. The villus height **(F)**, crypt depth **(G)**, and the ratio of villus height and crypt depth of jejunum **(H)**. The length **(I)**, histological damage score **(J)**, and crypt depth **(K)** of colon. All of the data are expressed as the mean ± SEM. Different superscript lowercase letters within each group indicate significantly different (*P* < 0.05).

No mortality was observed in the treatment groups during the experimental period. As shown in Figure 1A, group ETEC showed sustained body weight loss in comparison with the control group (*P* < 0.05). However, group ETEC + HNa showed weight loss trend during 1–3 days after infection and the weight gain was improved and had no difference with the group control (*P* > 0.05) from day 4. The spleen index (Figure 1B) of the ETEC group was higher than that of the control and ETEC + HNa group (*P* < 0.05). ETEC K88 infection significantly increased the diarrhea score (Figure 1C) as compared with the control group, while HNa treatment reversely decreased that (*P* < 0.05).

Figure 1D revealed that there was some damage to jejunal and colon villus after ETEC K88 infection, as found by broken and shortened villus. Consistent with the histological observations of tissue sections, ETEC K88 infection significantly reduced the VH (Figure 1F), and the ratio of VH and CD (Figure 1H) in the jejunum and increased histologic damage score (Figure 1J) and CD (Figure 1K) in the colon as compared with the control group, while HNa treatment reversely elevated that (*P* < 0.05). The jejunal CD (Figure 1G) and colon length (Figure 1I) did not differ (*P* > 0.05) among groups. It could be seen very intuitively that the intercellular adhesions and microvilli of jejunum and colon in the control group were compact, and arranged neatly, while the microvilli appeared disrupted and sparse after ETEC K88 infection. As expected, these ultrastructural changes were positively improved after HNa treatment (Figure 1E).

### Concentrations of Inflammatory Cytokines

**FIGURE 2:**
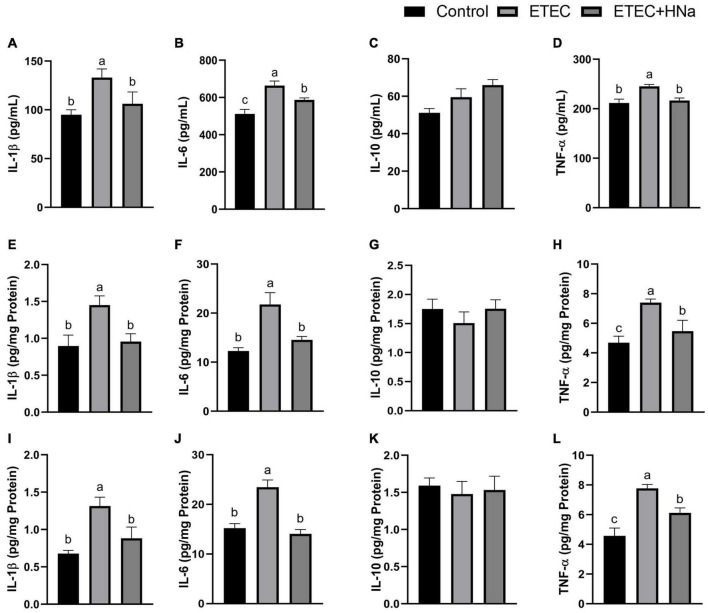
Effects of sodium humate on the concentrations of inflammatory cytokines in serum, jejunum, and colon of ETEC K88-infected mice. Concentrations of IL-1β, IL-6, IL-10, and TNF-α in the serum **(A–D)**, jejunum **(E–H)**, and colon **(I–L)**. All of the data are expressed as the mean ± SEM. Different superscript lowercase letters within each group indicate significantly different (*P* < 0.05).

To investigate whether HNa treatment could ameliorate inflammation caused by ETEC infection, we determined the concentrations of inflammatory cytokines in the serum, jejunum, and colon. The results revealed that ETEC K88 infection increased the concentrations of IL-1β, IL-6, and TNF-α in serum (Figures 2A,B,D), jejunum (Figures 2E,F,H), and colon (Figures 2I,J,L) when compared to control group (*P* < 0.05). As expected, these negative alterations were abrogated by HNa administration (*P* < 0.05). The concentration of IL-10 in the serum (Figure 2C), jejunum (Figure 2G), and colon (Figure 2K) were similar among groups (*P* > 0.05).

### Expression of Tight Junction Proteins and Mucosal Repair Factors

**FIGURE 3:**
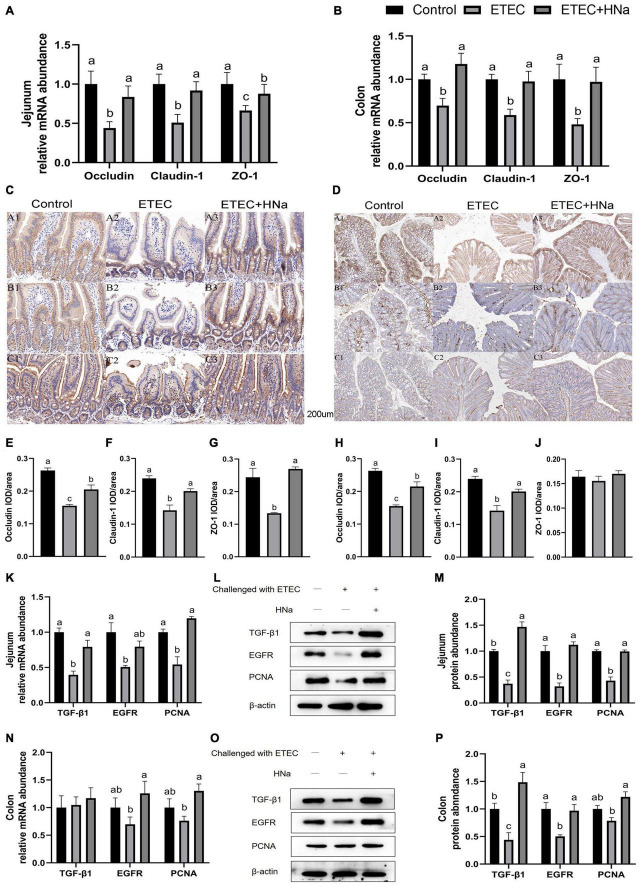
Effects of sodium humate on the mRNA and protein expression of tight junction proteins and intestinal epithelial cell repair factors in jejunum and colon of ETEC K88-infected mice. The mRNA expression of occludin, claudin-1, and ZO-1 in the jejunum **(A)** and colon **(B)**. Immunohistochemical staining of occludin, claudin-1, and ZO-1 in the jejunum **(C,E–G)** and colon **(D,H–J)** tissues (200 μm). The mRNA and protein expression of TGF-β1, EGFR, and PCNA in the jejunum **(K–M)** and colon **(N–P)** of mice. All of the data are expressed as the mean ± SEM. Different superscript lowercase letters within each group indicate significantly different (*P* < 0.05).

As shown in Figures 3A,B, ETEC K88 infection decreased the mRNA expression of occludin, claudin-1, and ZO-1 in the jejunum and colon compared with the control group (*P* < 0.05). In parallel with the lower mRNA expression levels of occludin, claudin-1, and ZO-1, ETEC K88 infection down-regulated protein levels of occludin (Figure 3E), claudin-1 (Figure 3F), and ZO-1 (Figure 3G) in the jejunum (Figure 3C, *P* < 0.05), and occludin (Figure 3H) and claudin-1(Figure 3I) in the colon (Figure 3D, *P* < 0.05). As expected, HNa treatment dramatically elevated the mRNA and protein levels of TJ proteins in the jejunum and colon (*P* < 0.05), indicating that HNa exerted protective effects on the intestinal barrier integrity.

To further verify the repair effects of HNa on the intestinal barrier of ETEC K88 infected mice, expression of epithelial cell proliferation-related factors was detected. The results revealed that mice in the ETEC + HNa group had higher mRNA expression of TGF-β1 and PCNA in the jejunum (Figure 3K) and EGFR and PCNA in the colon (Figure 3N), and higher protein expression of TGF-β1, EGFR, and PCNA in the jejunum (Figures 3L,M) and colon (Figures 3O,P) than mice in the ETEC group (*P* < 0.05).

### Intestinal Permeability

**FIGURE 4:**
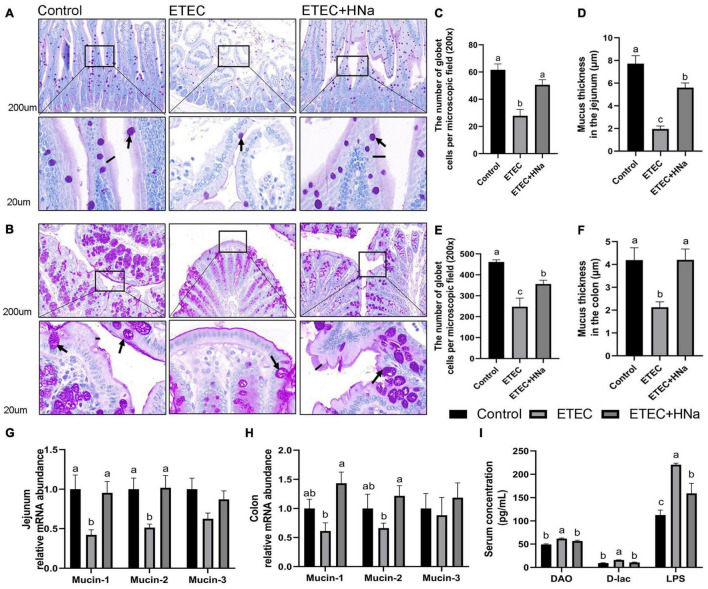
Effects of sodium humate on the intestinal permeability in jejunum and colon of ETEC K88-infected mice. Representative images of the jejunum **(A)** and colon **(B)** stained with PAS (200 μm). Arrow points to goblet cell; black bars indicate mucus layer thickness. The number of goblet cells per microscopic field and the mucus layer thickness in the jejunum **(C,D)** and colon **(E,F)** of mice. The relative mRNA expression of mucin-1, mucin-2, and mucin-3 in the jejunum **(G)** and colon **(H)** of mice. **(I)** The concentrations of serum DAO, D-lac, and LPS. All of the data are expressed as the mean ± SEM. Different superscript lowercase letters within each group indicate significantly different (*P* < 0.05).

The number of goblet cells (Figures 4C,E) and mucus thickness (Figures 4D,F). In the jejunum (Figure 4A) and colon (Figure 4B), and the mRNA expression of mucin-1 and mucin-2 in the jejunum (Figure 4G) were decreased after ETEC K88 infection as compared with controls (*P* < 0.05). Nevertheless, the decline in the ETEC group was reversed to normal values by HNa treatment (*P* < 0.05). Serum DAO, D-lac, and LPS concentration are important indicators for intestinal barrier injury. As shown in Figure 4I, the ETEC K88 infected mice showed increased concentrations of serum DAO, D-lac, and LPS compared with the control and ETEC + HNa groups (*P* < 0.05), indicating that ETEC K88 infection resulted in severe intestinal barrier injury.

### Intestinal Microbiota Composition

**FIGURE 5:**
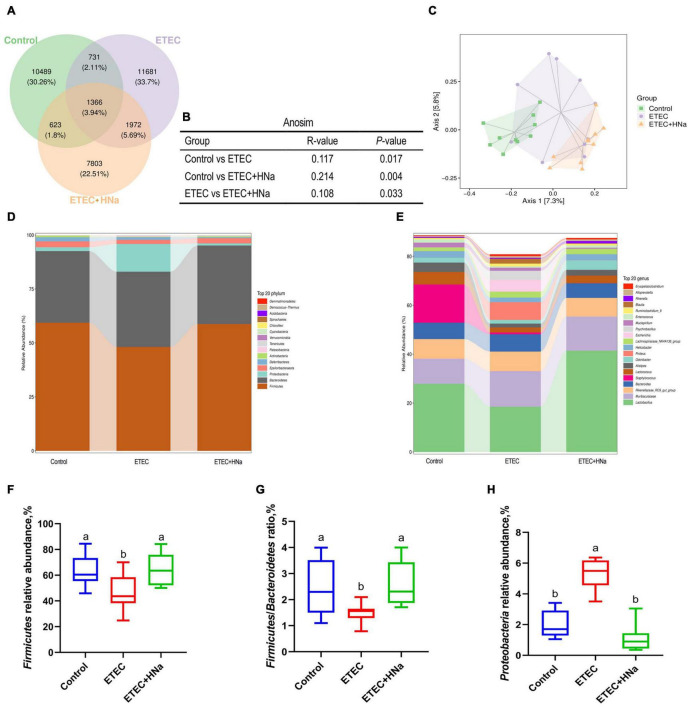
Effects of sodium humate on intestinal microbiota composition of ETEC K88-infected mice. **(A)** A Venn diagram displayed the overlaps among groups (*n* = 10). **(B)** Anosim analysis among groups. **(C)** Principal co-ordinates analysis (PCoA) plot of intestinal microbiota; each represented by one color (*n* = 10). Community bar plots of phylum and genus level were shown in **(D,E)**. **(F)** The relative abundance of *Firmicutes*. **(G)** The ratio of *Firmicutes* to *Bacteroidetes*. **(H)** The relative abundance of *Proteobacteria*. All of the data are expressed as the mean ± SEM. Different superscript lowercase letters within each group indicate significantly different values (*P* < 0.05).

To reveal the impact of HNa on the intestinal microbiota community of mice under ETEC K88 infection, we conducted the analysis of the V3–V4 region of 16S rDNA gene sequences of microbiota in feces. After denoising using DADA2, an average of 96,329 16S rRNA gene sequences per sample was obtained, and 38,169 amplicon sequence variants (ASVs) were obtained from 30 samples after filtering. Moreover, a Venn diagram (Figure 5A) showed that there were 1,366 shared ASVs among three groups, and 10,489, 11,681, and 7,803 unique ASVs were observed in control, ETEC, and ETEC + HNa groups, respectively. The Chao1, Ace, Shannon, Simpson indexes, and observed species associated with bacterial richness and diversity were similar among groups (Supplementary Figures 1A–E; *P* > 0.05). The plots of principal coordinate analysis (PCoA) (Figure 5C) and Anosim (Figure 5B) showed distinct clustering of the microbial community for each group.

The relative abundances of different phyla were shown in Figure 5D. The microbial community was dominated by *Firmicutes*, *Bacteroidetes*, *Proteobacteria*, and *Epsilonbacteraeota*, which were more than 97%. We found that ETEC K88 infection decreased the relative abundance of *Firmicutes* and *Firmicutes*/*Bacteroidetes*, and increased the relative abundance of *Proteobacteria* (*P* < 0.05) whereas HNa treatment reversed that (Figures 5F–H). At the genus level (Figure 5E), the intestinal microbiota was dominated by *Lactobacillus*, *Muribaculaceae*, *Rikenellaceae RC9 gut group*, *Bacteroides*, *Staphylococcus*, *Lactococcus*, *Alistipes*, *Odoribacter*, *Proteus*, and *Helicobacter*.

**FIGURE 6:**
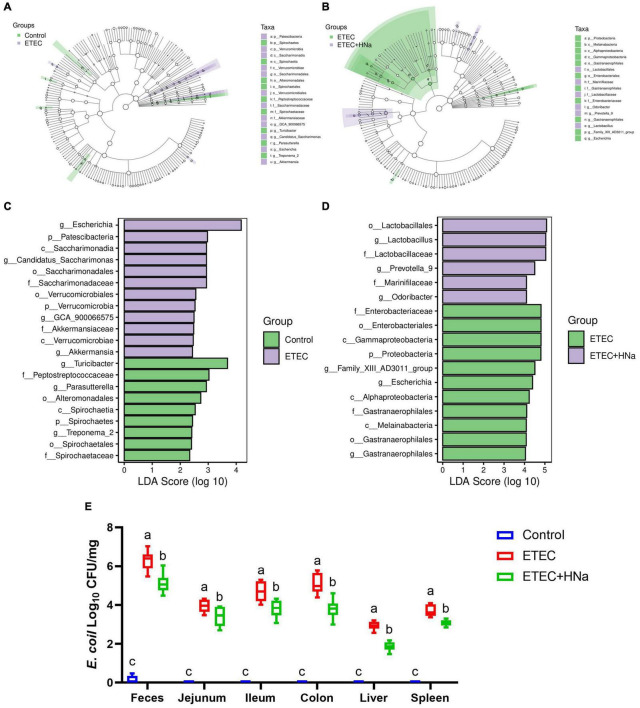
Effect of sodium humate on the diversity of intestinal microbiota of ETEC K88-infected mice. Control vs ETEC **(A)** and ETEC vs ETEC + HNa **(B)** groups taxonomic cladogram of LEfSe analysis. Different colors indicate the enrichment of the biomarker taxa in different groups. The circle from inside to outside means the rank from kingdom to species, and the circle size represents the taxa abundance in the community. Control vs ETEC **(C)** and ETEC vs ETEC + HNa **(D)** linear discriminant analysis (LDA) scores for different taxa abundances. **(E)** The number of *E. coli* in jejunal mucosa, ileal mucosa, colonic mucosa, feces, liver, and spleen (*n* = 10). All of the data are expressed as the mean ± SEM. Different superscript lowercase letters within each group indicate significantly different (*P* < 0.05).

To find the key phylotypes and biomarkers of intestinal microbiota among various groups, the LEfSe and LDA analysis were performed (Figures 6A–D). Among the cross-comparisons of different groups (control vs ETEC, ETEC vs ETEC + HNa), LDA results showed 12 discriminative features at the genus level. As presented in Figure 6C, the abundances of *Escherichia*, *GCA_900066575*, *Candidatus_Saccharimonas*, and *Akkermansia* were enriched in the ETEC group compared with the control group (*P* < 0.05). In contrast to the ETEC group, HNa treatment increased (*P* < 0.05) the abundances of *Lactobacillus*, *Prevotella_9*, and *Odoribacter* while decreased the abundances of *Escherichia* and *Gastranaerophilales* (Figure 6D).

Linear discriminant analysis analysis indicated that the abundance of *Escherichia* was enriched in the ETEC group compared with the control and ETEC + HNa groups. To probe the inhibitory effect of HNa on *E. coli*, we investigated the number of *E. coli* in the feces, intestinal, liver, and spleen of mice. As shown in Figure 6E, compared with the control group, ETEC K88 infection spiked the number of *E. coli* in the feces, ileum, jejunum, colon, liver, and spleen (*P* < 0.001). However, the administration of HNa substantially reduced the number of *E. coli* (*P* < 0.05), indicating that HNa had strong antimicrobial activity.

### Analysis of Fecal Metabolites

**FIGURE 7:**
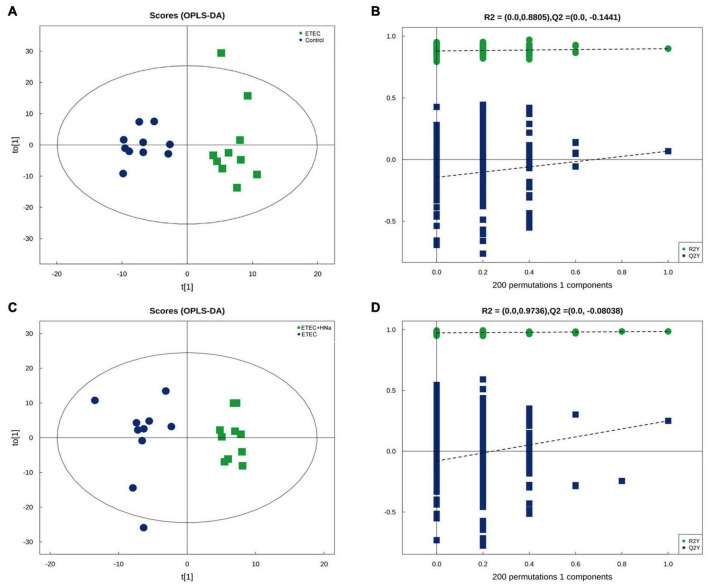
Differentiation of the metabolic profiles using multivariate analysis. Orthotopic partial least-squares discriminant analysis (OPLS-DA) score plots from control vs ETEC **(A,B)** and ETEC vs ETEC + HNa **(C,D)** of fecal metabolites under negative ion mode. R2 and Q2 represent the interpretability and predictability of models. Enriched Kyoto Encyclopedia of Genes and Genomes (KEGG) pathway enrichment based on altered metabolites. The color of the point was *P*-value, and the redder, the more significant enrichment. The size of the spot represented the number of different metabolites enriched. Control vs ETEC group **(E)**; ETEC + HNa vs ETEC group **(F)**.

In the present study, we further examined the fecal metabolic profiles via untargeted metabolomics analysis in order to determine whether the beneficial effect of HNa might be attributed to metabolites alterations. There were overall 329 and 234 metabolites identified in the feces of mice, under positive and negative ion mode, respectively. The OPLS-DA plots showed a clear separation for control vs ETEC (Figure 7A) and ETEC vs ETEC + HNa (Figure 7C) in the negative ion model, indicating that the feces metabolic profiles of mice were distinctly different among varied groups. The R2 and Q2 of OPLS-DA score analysis indicate that models were valid (Figures 7B,D).

Metabolites with VIP values > 1.0 and *P*-value < 0.05 were considered significantly different. As shown in Table 1 according to multivariate statistical analysis, a total of eight (Hydroxyacetone and alpha-Tochopheryl Acetate significantly upregulated and 4-Pyridoxic acid, Palmitic acid, 2E-Eicosenoic acid, Tetrahydro-L-biopterin, Tyr-Lys, and alpha-Linolenic acid significantly downregulated) significantly changed metabolites in fecal samples of mice were detected in the control group compared with the ETEC group. Additionally, a total of 15 (3-Phenylpropanoic acid, Isobutyric acid, Gentisic acid, Deoxycytidine, Trp-Arg, alpha-Linolenic acid, and Propionic acid significantly upregulated and Erucamide, 1-Stearoyl-rac-glycerol, 2-Ethoxyethanol, Val-Asp, Sphinganine, Chenodeoxycholate, Succinate, and 1,3,5(10)-Estratrien-3,17.beta.-diol17-glucosiduronate significantly downregulated) significantly changed metabolites were detected in the ETEC + HNa group compared with the ETEC group (Table 2).

**TABLE 1:** Identified significant different metabolites from control and Enterotoxigenic *Escherichia coli* (ETEC) groups under positive and negative ion model^a^.

Adduct	Metabolites	VIP	FC	*P*-value	m/z	rt (s)
(M + Na)+	Tyr-Lys	1.06	0.51	0.008	332.15	425.21
(M + NH4)+	alpha-Tochopheryl Acetate	3.23	2.09	0.009	490.42	32.00
(M + Na)+	Tetrahydro-L-biopterin	2.31	3.08	0.02	264.10	165.08
(M + NH4)+	alpha-Linolenic acid	3.68	0.85	0.03	296.25	36.71
(M − H)−	4-Pyridoxic acid	5.59	1.79	0.02	182.04	41.65
(M + CH3COO)−	Hydroxyacetone	2.53	0.28	0.04	133.04	202.18
(M − H)−	Palmitic acid	9.33	1.75	0.04	255.23	44.7
(M − H)−	2E-Eicosenoic acid	2.73	1.55	0.04	309.27	39.28

*Difference metabolites identified by positive and negative ion mode (multi-dimensional statistical analysis of VIP > 1 and univariate statistical analysis of P-value < 0.05), the control group versus the ETEC group.*

*^a^Adduct, adduct ion information of the compound; VIP, variable importance in the projection; FC, Fold change; m/z, mass-to-charge ratio; Rt(s), retention time.*

**TABLE 2:** Identified significantly different metabolites from Enterotoxigenic *Escherichia coli* (ETEC) and ETEC + Sodium humate (HNa) groups under positive and negative ion model^a^.

Adduct	Metabolites	VIP	FC	*P*-value	m/z	rt (s)
(M + H − H2O)+	1-Stearoyl-rac-glycerol	1.18	0.51	0.004	341.30	191.95
(M + CH3COO + 2H)+	2-Ethoxyethanol	1.18	0.78	0.008	151.09	65.48
(M + H)+	Trp-Arg	1.01	0.57	0.03	361.19	339.80
(M + H)+	Erucamide	1.69	1.44	0.03	338.34	34.44
(M + H − H2O)+	Val-Asp	2.63	0.25	0.03	215.10	342.13
(M + NH4)+	alpha-Linolenic acid	2.95	0.84	0.0	296.25	36.71
(M + H)+	Deoxycytidine	3.19	2.29	0.042	228.0	205.20
(M + H)+	Sphinganine	1.51	0.58	0.044	302.30	109.24
(M + CH3COO)−	Chenodeoxycholate	3.14	0.60	0.02	451.30	154.48
(M − H)−	Propionic acid	5.76	0.47	0.03	73.02	383.06
(M − H)−	Succinate	8.86	0.47	0.03	117.01	383.06
(M − H)−	Gentisic acid	1.68	1.72	0.03	153.01	70.10
(M − H)−	3-Phenylpropanoic acid	6.68	1.94	0.03	149.06	98.09
(M − H)−	17-glucosiduronate	1.25	0.43	0.03	447.20	127.10
(M − H)−	Isobutyric acid	11.46	1.81	0.04	87.04	122.35

*Different metabolites identified by positive and negative ion mode (multi-dimensional statistical analysis of VIP > 1 and univariate statistical analysis of P-value < 0.05), the ETEC + HNa group versus the ETEC group.*

*^a^Adduct, adduct ion information of the compound; VIP, variable importance in the projection; FC, Fold change; m/z, mass-to-charge ratio; Rt(s), retention time.*

**FIGURE 8:**
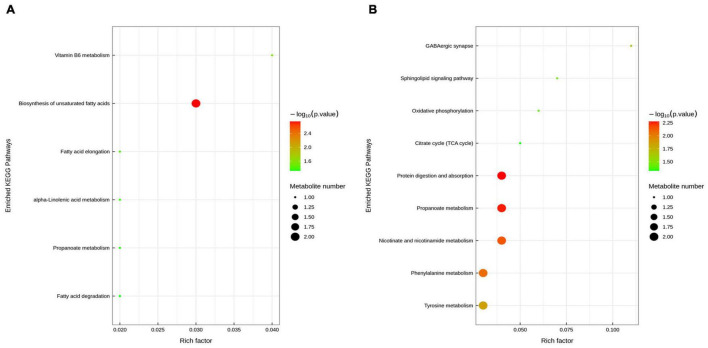
Enriched Kyoto Encyclopedia of Genes and Genomes (KEGG) pathway enrichment based on altered metabolites. The color of the point was *P*-value, and the redder, the more significant enrichment. The size of the spot represented the number of different metabolites enriched. Control vs ETEC group **(A)**; ETEC + HNa vs ETEC group **(B)**.

To reveal the underlying mechanism, these changed metabolites were further performed by KEGG enrichment analysis. As shown in Figure 8A, biosynthesis of unsaturated fatty acids, vitamin B6 metabolism, fatty acid elongation, alpha-Linolenic acid metabolism, propanoate metabolism, and fatty acid degradation were significantly enriched in ETEC group compared with control group (*P* < 0.05). As shown in Figure 8B, protein digestion and absorption, propanoate metabolism, nicotinate and nicotinamide metabolism, phenylalanine metabolism, tyrosine metabolism, GABAergic synapse, sphingolipid signaling pathway, oxidative phosphorylation, and citrate cycle (TCA cycle) were markedly enriched (*P* < 0.05) in ETEC + HNa group compared with ETEC group. By cross-comparisons of control vs ETEC and ETEC vs ETEC + HNa group, both control and ETEC + HNa group have more enrichment on the biosynthesis of unsaturated fatty acids and propanoate metabolism.

### Correlation Analysis of Microbiota and Metabolites

**FIGURE 9:**
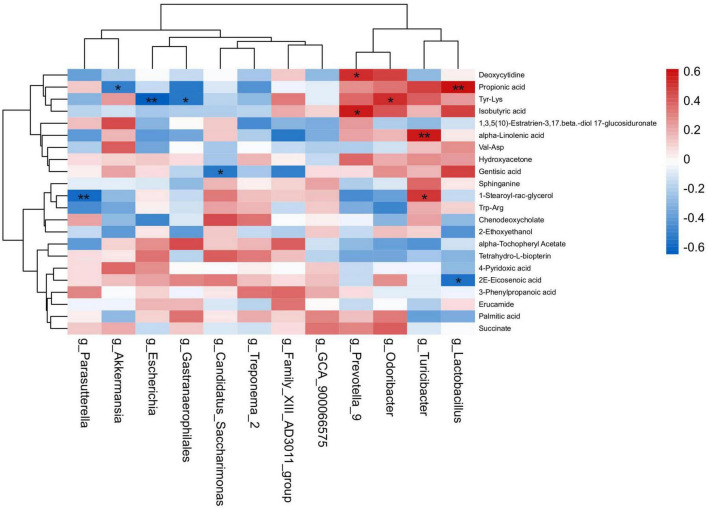
Correlation analysis of the intestinal microbiota and metabolites. Spearman’s rank correlation between eight most differential genera and 22 differential metabolites. *P < 0.05, **P < 0.01 denoted statistical significance between microbiota and metabolites.

The spearman’s correlation analysis was conducted to identify the relationship of differential fecal microbiota in genus level and differential metabolites upon HNa treatment (Figure 9). In detail, *g_Lactobacillus* was positively correlated with Propionic acid (*r* > 0.50, *P* < 0.01) and negatively correlated with 2E-Eicosenoic acid (*r* > 0.50, *P* < 0.05). *g_Turicibacter* was positively correlated with alpha-Linolenic acid and 1-Stearoyl-rac-glycerol (*r* > 0.50, *P* < 0.05). *g_Gastranaerophilales* was negatively correlated with Tyr-Lys (*r* > 0.50, *P* < 0.05). *g_Odoribacter* was positively correlated with Tyr-Lys (*r* > 0.50, *P* < 0.01). *g_Prevotella_9* was positively correlated with Isobutyric acid and Deoxycytidine (*r* > 0.50, *P* < 0.01). *g_Escherichia* was negatively correlated with Tyr-Lys (*r* > 0.50, *P* < 0.01). *g_Candidatus_Saccharimonas* was negatively correlated with Gentisic acid (*r* > 0.50, *P* < 0.05). *g_Parasutterella* was negatively correlated with 1-Stearoyl-rac-glycerol (*r* > 0.50, *P* < 0.01). *g_Akkermansia* was negatively correlated with Propionic acid (*r* > 0.50, *P* < 0.05).

## Discussion

Enterotoxigenic *Escherichia coli*, a common environmental pathogen, can result in acute watery diarrhea accompanied by fever, vomiting, and dehydration in newborns and young animals (Ledwaba et al., 2020). Numerous studies elucidated that ETEC K88 infection could cause great economic loss in livestock industry due to poor growth performance and high mortality of animals (Qiu et al., 2017; Becker et al., 2020; Wang Q. et al., 2020). In the present study, we investigated the protective potential of HNa against ETEC K88 infection in mice. The results revealed that the BW of mice was decreased but the spleen index and diarrhea score was increased after ETEC K88 infection, while HNa treatment reversed these changes. Accordingly, we found that ETEC K88 infection reduced the number of goblet cells, mucus thickness, and mRNA expression of mucin-1 and mucin-2, and the mRNA and protein expression of TJ proteins in the jejunum and colon of mice. Additionally, the concentrations of serum DAO, D-lac, and LPS were distinctly increased in accordance with the down-regulated levels of TJ proteins and mucins. Similarly, the reduced VH and VH/CD in jejunal tissue and increased histologic damage score and CD in colon tissue were observed after ETEC K88 infection in the present study. These results showed that ETEC K88 infection resulted in severe intestinal injury. However, therapeutic administration of HNa effectively restored the intestinal barrier integrity and maintained the normal ratio of villus-crypt. Intestinal integrity is vital to maintain the host’s innate immunity, which could prevent intestine damage from pathogenic bacteria, LPS, and toxins (Johansson et al., 2011). Thus, the integrity of intestinal barrier is essential for maintaining intestinal function. Previous studies reported that intestinal permeability was significantly increased in mice (Ledwaba et al., 2020) and piglets (Becker et al., 2020) after ETEC infection. The intestinal barrier function is associated with the expression of TJ proteins including ZO-1, claudin-1 and occludin, and mucins (Liang et al., 2020). The mucus layer is formed by mucin-1, mucin-2, and mucin-3, primarily mucin-2 in the small intestine and colon, which is secreted by goblet cells. The mucus layer covering the surface of the intestinal epithelial cells could protect the intestinal epithelial barrier integrity (Liang et al., 2020). It was reported that *E. coli* adhered to intestinal epithelial cells could damage and thin the mucus layer, decrease the expression of TJ proteins, and increase intestinal permeability in mice (Zou et al., 2020). Previous studies also reported that the decreased mucus layer thickness was associated with pathogenic infection (Yi et al., 2017; Furter et al., 2019). In this study, we found that ETEC K88 infection reduced the number of goblet cells, mRNA expression of mucin-1 and mucin-2, and the mRNA and protein expression of TJ proteins in the jejunum and colon of mice. Interestingly, HNa treatment effectively attenuated the ETEC K88-induced reduction in mucus layer thickness in the jejunum and colon. We surmise that the increased thickness of mucus layer may be related to the increased numbers of goblet cells.

Inflammatory responses could affect the expression of TJ proteins and mucins, and the expression of TJ proteins is always decreased in patients with enteritis (Mees et al., 2009). Upon *E. coli* attachment to the intestinal mucosa, immune cells are activated to produce cytokines, which play an important role in regulating the inflammatory process. It was reported that pro-inflammatory factors such as IL-1β, IL-6, and TNF-α are important indicators in response to ETEC K88 (Qiao et al., 2020). The present study found that ETEC K88 infection dramatically increased the concentrations of IL-1β, IL-6, and TNF-α in the serum, jejunum, and colon of mice. Studies have revealed that TNF-α plays an important role in the mediation of occludin internalization, and the occludin has a positive relationship with intestinal permeability. The overexpression of TJ proteins could ameliorate cytokine-induced increases in intestinal permeability (Marchiando et al., 2010). In the enteritis patient, TNF-α and IL-1β not only increase the infiltration of neutrophils, but also cause damage to the intestinal barrier (Cao et al., 2004). Cytokines such as IL-1β and IL-6 can also decrease the expression of TJ proteins through the adenosine monophosphate-activated protein kinase dependent pathway (Scharl et al., 2009). Moreover, cytokines cause myosin light-chain phosphorylation, which could damage the TJ proteins. Then the pathogenic bacteria or/and LPS could enter the intestine through the damaged intestinal barrier, which could bind to toll-like receptors and stimulate the TLR-mediated signaling pathway, further aggravate the inflammatory response (Masaki et al., 2018). As expected, HNa administration effectively inhibited the production of pro-inflammatory cytokines in ETEC K88 infected mice. Collectively, these data suggest that HNa can maintain the intestinal barrier integrity via improving the thickness of the mucus layer, expression of TJ proteins, and inhibiting the production of pro-inflammatory cytokines in the jejunum and colon of ETEC K88 infected mice. Similar with our results, Brandts et al. (2021) revealed that the inclusion of HNa exerts a remarkable anti-inflammatory effect in European seabass. HNa is rich in active groups such as phenolic-hydroxyl, carboxyl, sulfhydryl, and carbonyl which may contribute to its anti-inflammatory properties (Xu et al., 2020).

In addition, numerous studies have revealed the positive effects of PCNA, EGFR, and TGF-β1 on epithelial cell proliferation, migration, and angiogenesis (Fernández Lorente et al., 2013; Dkhil et al., 2016; Ji et al., 2016). Surprisingly, HNa treatment elevated the mRNA and protein expression of TGF-β1, PCNA, and EGFR in the jejunum and colon of mice, indicating that HNa could restore the damaged intestinal epithelium via promoting epithelial proliferation. The serum levels of DAO, D-lac, and LPS are important markers for intestinal epithelial barrier function. Our results indicated that HNa treatment reduces the serum levels of DAO, D-lac, and LPS, which further confirmed that HNa could maintain the intestinal barrier integrity in ETEC K88 infected mice. Furthermore, intestinal barrier damage can cause intestinal bacteria transfer (Ling et al., 2016). Given the protective effect of HNa on the intestinal epithelial barrier, we further investigated the translocation of intestinal bacteria in tissues of mice. We consistently found that *E. coli* were more existed in the liver and spleen of mice in the ETEC group than that in the HNa-treated group, suggesting that HNa-treated mice can defend against the intestinal *E. coli* translocation by decreasing intestinal permeability. Consistent with our results, an earlier study showed that dietary HNa supplementation decreased the *E. coli* adhesion and colonization in the gut of ETEC infected piglets, indicating the underlying inhibitory effects of HNa on pathogen invasion (Hu et al., 2020). Taken together, HNa administration had protective effects on intestinal barrier integrity of the ETEC K88 infected mice via increasing TJ proteins and mucins expression, promoting enterocyte proliferation, and reducing the release of inflammatory cytokines.

Intestinal microbiota plays a key role in maintaining intestinal homeostasis, regulating intestinal immune response, and protecting the host against pathogens (Fujiwara et al., 2018). The first step of infection by pathogenic bacteria is to adhere to and colonize the intestinal tract, and the number of adhesions directly reflects the infection situation. Thus, we investigated the number of *E. coli* colonization in the jejunum, ileum, and colon of mice and found that the administration of HNa significantly reduced the number of *E. coli* colonization compared with the mice in the ETEC group. Previous studies revealed that *E. coli* infection could disturb intestinal microbiota (Wang Y. et al., 2020; Zou et al., 2020). It was reported that dietary supplementation of HNa altered the microbial composition and reduced the abundance of pathogenic *E. coli* in ETEC infected piglets (Kaevska et al., 2016). In agreement with the study, we found that the microbiota community of ETEC group was markedly separate from that of the control and ETEC + HNa groups. The ratio of *Firmicutes*/*Bacteroidetes* is considered to be an important indicator for maintaining the balance of intestinal microflora (Elokil et al., 2020). ETEC K88 infection decreased the relative abundance of *Firmicutes* and ratio of *Firmicutes*/*Bacteroidetes*, while the relative abundance of *Proteobacteria* was increased. HNa treatment positively altered these changes. This observation indicated that HNa could maintain intestinal microflora homeostasis. Additionally, LDA analysis was performed to identify the differential bacteria at the genus level among groups. *Escherichia* is a conditional pathogen, which produces Shiga toxin during infection and causes intestinal inflammation. Wei et al. (2020) found that *E. coli* infection increased the colonization of pathogenic bacteria and decreased the beneficial bacterial flora in the gut of mice. This is similar to our results that the abundances of *Escherichia* and *Saccharimonas* were enriched in mice challenged with ETEC K88. Increased beneficial bacteria can compress the living space and nutrition supply of harmful bacteria, thereby reduce colonization by pathogens. Importantly, the present study indicated that HNa treatment not only decreased the abundance of *Escherichia*, but also increased the abundance of beneficial bacteria, such as *Lactobacillus*, *Prevotella_9*, and *Odoribacter*. Furthermore, we further investigated the number of *E. coli* in the feces of mice and found that the administration of HNa significantly reduced the number of *E. coli* compared with the mice in ETEC group. Similarly, our previous study revealed that dietary supplementation with HNa increased the abundances of *Bifidobacterium* and *Lactobacillus* and reduced the abundance of *E. coli* in weaned calves (Wang et al., 2021). It is well known that *Lactobacillus* could improve intestinal health by inhibiting colonization of pathogens and enhancing mucosal immunity (Qiao et al., 2020). *Prevotella* and *Odoribacter*, which are anaerobic gram-negative bacterium, can produce short-chain fatty acids (SCFAs), modulate inflammatory responses, and exert an anti-inflammatory effect in patients with inflammatory bowel disease (Li et al., 2021). It was reported that intestinal *Prevotella* are key players in host-microbiome interactions, especially in relation to nutrition and metabolism (Tett et al., 2021). Moreover, Xing et al. (2021) reported that *Odoribacter* can promote intestinal Th17 cell proliferation and protect host against colitis and colorectal cancer in wild-type mice. These studies further confirmed our results that HNa had beneficial effects on maintaining intestinal microbiota homeostasis. Studies have shown that HNa has an inhibitory effect on the proliferation of *E. coli in vivo* and *vitro*. The results of present study also indicated that the abundance of *Escherichia* was significantly lower in mice treated with HNa. In summary, the altered intestinal microbiota abundance of HNa administration may be related to its inhibition effects on pathogenic bacteria such as *E. coli*. Moreover, it was reported that changes in intestinal microbiota are closely associated with intestinal barrier function (Bai et al., 2021). Thus, we speculate that HNa may alleviate intestinal dysfunction induced by ETEC K88 through maintaining intestinal homeostasis and increasing the abundance of intestinal beneficial bacteria.

Intestinal microflora performs critical metabolic, trophic, and protective functions, and the fecal metabolome can be detected to assess microbial function (Yang et al., 2019). The profiles and types of intestinal microflora could influence the production of relevant metabolic products, which are absorbed into the circulation and eventually metabolized by the host. For instance, polysaccharides can be metabolized by the intestinal microbiota and finally fermented into SCFAs which modulate the intestinal microbiota and promote intestinal epithelial cell proliferation (Shao et al., 2019). Bittinger et al. (2020) revealed that the appearance of microflora was associated with an increase in intestinal fermentation products. In addition, infant meconium metabolomics also reveals that metabolites are differentially abundant features with microbial colonization. Thus, the disordered intestinal microflora can further influence intestinal metabolic profiles (Zhou et al., 2021). Our results found that the mice in ETEC group showed significantly decreased levels of unsaturated fatty acids compared with the control group. Unsaturated fatty acids are essential for maintaining intestinal health and immune function (Bogataj Jontez et al., 2021). For example, alpha-Linolenic acid has anti-inflammatory and neuroprotective properties (Litwiniuk et al., 2020). It is worth noting that HNa treatment enriched some metabolites associated with immunoregulation and cells proliferation. For example, propionic acid, one of the SCFAs, could promote intestinal epithelial cell regeneration and activate intestinal mucosal immunity under inflammatory conditions (Naß et al., 2021). Isobutyric acid and gentisic acid possess anti-inflammatory and antimicrobial activities (Huang et al., 2021). The regulatory effect of Trp-Arg on key immunological processes was widely known (Li et al., 2019). Gut microflora-mediated conversion plays a crucial role in modulating drug metabolism. Gut metabolism was considered to be one of the main factors contributing to the pronounced pharmacological effects of drugs. Ai et al. (2021) found that coptisine was transformed into 8-oxocoptisine (OCOP) by intestinal microbiota, and exhibited appreciable anti-inflammatory, anti-tumor, anti-fungal, and cardiovascular-protective effects, and revealed the importance of the intestinal microbiota in enhancing the therapeutic efficacy of natural products. Importantly, spearman’s correlation analysis found that enriched metabolites were positively correlated with increased beneficial intestinal microbiota in ETEC + HNa group. Taken together, all of the above findings provide evidences that intestinal microbiota is associated with metabolites and the disruption of intestinal microbiota can lead to dysregulated homeostasis of host metabolism. In the present study, ETEC K88 infection detrimentally altered intestinal microbiota composition and metabolic profiles in mice, whereas HNa treatment positively improved that.

## Conclusion

In summary, HNa administration effectively alleviated adverse effects of ETEC K88 infection by enhancing epithelial barrier integrity, suppressing inflammatory responses, and modulating intestinal microbiota and metabolites. HNa might be an effective and safe therapeutic agent for ETEC infection.

## Data Availability Statement

The data presented in the study are deposited in the National Center for Biotechnology Information (NCBI) Sequence Read Archive (SRA) repository, accession number PRJNA799167.

## Ethics Statement

The experimental protocol was approved by the Ethics Committee of Northeast Agricultural University (Harbin, China).

## Author Contributions

DW: writing-original draft, formal analysis, and investigation. YH and KL: assisted in animal feeding and laboratory work. SD and YF: conducted research and data collection. YL: project administration, editing, validation, and funding acquisition. All authors read and approved the final manuscript.

## Conflict of Interest

The authors declare that the research was conducted in the absence of any commercial or financial relationships that could be construed as a potential conflict of interest.

## Publisher’s Note

All claims expressed in this article are solely those of the authors and do not necessarily represent those of their affiliated organizations, or those of the publisher, the editors and the reviewers. Any product that may be evaluated in this article, or claim that may be made by its manufacturer, is not guaranteed or endorsed by the publisher.

## Footnotes

1
http://www.genome.jp/kegg
