# Supplementary material for: Sodium Humate Alleviates Enterotoxigenic Escherichia coli-Induced Intestinal Dysfunction via Alteration of Intestinal Microbiota and Metabolites in Mice
Source: Front Microbiol. 2022 Mar 25;13:809086. doi: 10.3389/fmicb.2022.809086 (PMC8992542; doi:10.3389/fmicb.2022.809086)
Supplement: Supplementary file 1 [file Data_Sheet_1.PDF]

# *Supplementary Material*

## 1 Supplementary Table

**Supplementary Table 1.** Primer sequences used for quantitative real-time PCR.

| **Primer**[1] | **Sequence (5'→3')** | **Product size, bp** | **GeneBank accession No.** |
|---|---|---|---|
| Mucin-1 | F: GGCATTCGGGCTCCTTTCTT<br>R: TGGAGTGGTAGTCGATGCTAAG | 132 | NP_038633 |
| Mucin-2 | F: AGGGCTCGGAACTCCAGAAA<br>R: CCAGGGAATCGGTAGACATCG | 106 | AJ511872 |
| Mucin-3 | F: CCGAGAGCGGAAGTGTGTG<br>R: TGTAACTGTGGTTTTGGTCTTCA | 101 | AF027131 |
| TGF-β1 | F: CTCCCGTGGCTTCTAGTGC<br>R: GCCTTAGTTTGGACAGGATCTG | 133 | NM_011577 |
| PCNA | F: TTTGAGGCACGCCTGATCC<br>R: GGAGACGTGAGACGAGTCCAT | 135 | NM_011045 |
| EGFR | F: GCCATCTGGGCCAAAGATACC<br>R: GTCTTCGCATGAATAGGCCAAT | 101 | NM_207655 |
| Occludin | F: TTGAAAGTCCACCTCCTTACAGA<br>R: CCGGATAAAAAGAGTACGCTGG | 129 | NM_008756 |
| Claudin-1 | F: GGGGACAACATCGTGACCG<br>R: AGGAGTCGAAGACTTTGCACT | 100 | NM_016674 |
| ZO-1 | F: GCCGCTAAGAGCACAGCAA<br>R: TCCCCACTCTGAAAATGAGGA | 134 | NM_0011635<br>74 |
| β-actin | F: GGCTGTATTCCCCTCCATCG<br>R: CCAGTTGGTAACAATGCCATGT | 154 | NM_007393 |

[1]ZO-1: Zona occludens 1; TGF-β1: transforming growth factor-β1; PCNA: proliferative cell nuclear antigen; EGFR: epidermal growth factor receptor.
F: Forward primer; R: Reverse primer.

### 1.1 Supplementary Figures

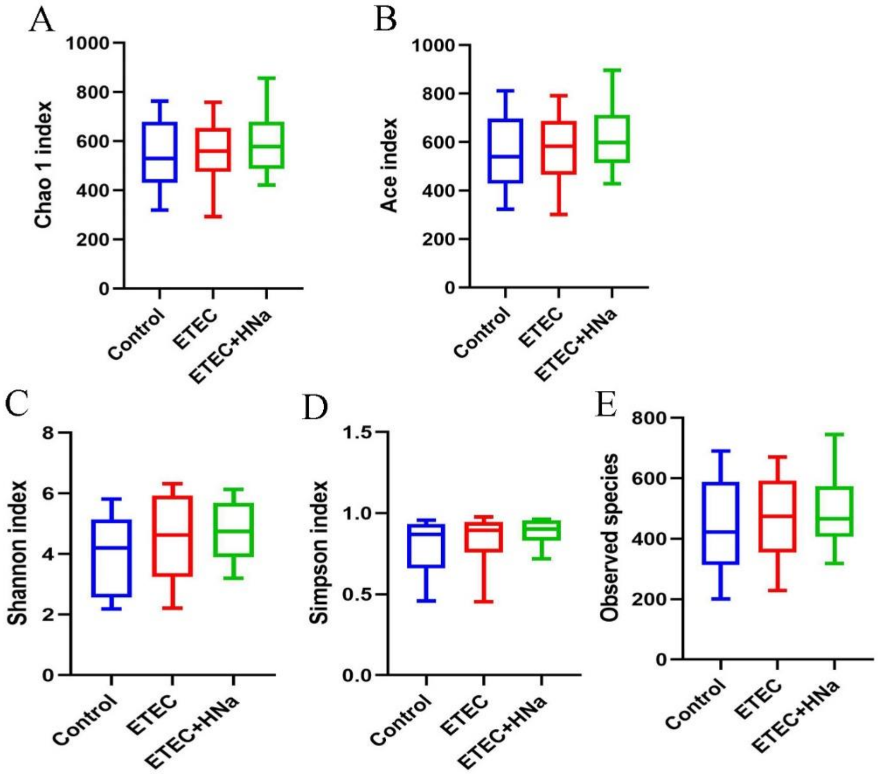


**Supplementary Figure 1.** The results of intestinal microbiota alpha diversity analysis and significant differences at the phylum level among groups. (**A-E**) Chao 1, Ace, Shannon, Simpson index, and Observed species.
